# Wide-field ESD for Barrett's adenocarcinoma at the gastroesophageal junction: technical approaches to facilitate en bloc R0 resection

**DOI:** 10.1016/j.vgie.2022.08.004

**Published:** 2022-10-01

**Authors:** Fabian Emura, Manuel Arrieta-Garcia, Raúl Castilllo-Delgado, Huber Padilla-Zambrano

**Affiliations:** 1 Gastroenterology Division, Universidad de La Sabana, Chía, Colombia; 2 Endoscopy, Clínica Imbanaco, Grupo Quironsalud, Cali, Colombia; 3 Advanced GI Endoscopy, EmuraCenter LatinoAmerica, Bogotá DC, Colombia; 4 Pathology, Clínica Imbanaco, Grupo Quironsalud, Cali, Colombia

**Keywords:** BA, Barrett adenocarcinoma, BE, Barrett's esophagus, ESD, endoscopic submucosal dissection, GEJ, gastroesophageal junction, IM, intestinal metaplasia, RFA, radiofrequency ablation, SM, submucosal, WF, wide-field

## Abstract

Video 1 Wide-field ESD for Barrett's adenocarcinoma.

Wide-field ESD for Barrett's adenocarcinoma.

## Background

Endoscopic submucosal dissection (ESD) for Barrett's esophagus (BE) neoplasia is associated with high en bloc resection and an acceptable safety profile but with suboptimal curability rates (range, 56%-59%).^1^ Two large Western studies showed that low R0 resection rates resulted from the high rate of positive lateral margins in ESD specimens (range, 82%-86%), which prompted either salvage ESD or additional surgical treatment.^2^^,^^3^ A U.S. multicenter ESD study found positive lateral margins in up to 70% of Barrett's adenocarcinoma (BA) at the gastroesophageal junction (GEJ) because of the increased technical complexity, poor maneuverability, and difficulty in evaluating the lesion’s margin.^4^ Recently, ESD with wider resection margins (5-10 mm) has been proposed as an alternative to increasing the R0 resection rate and, therefore, the curability of Barrett's neoplasia.^4^ In a comparative study of wide-field (WF) ESD versus conventional ESD, WF-ESD resulted in a significantly higher curability rate, with fewer positive lateral margins and lower metachronous recurrences compared to conventional ESD.^5^ Technical approaches to reduce the positivity of lateral margins of ESD for BA located at the GEJ have not been described. We present a video case of WF-ESD for a BA of the GEJ and describe novel approaches for facilitating en bloc R0 resections (Video 1, available online at www.giejournal.org).

## Case

**Figure 1:**
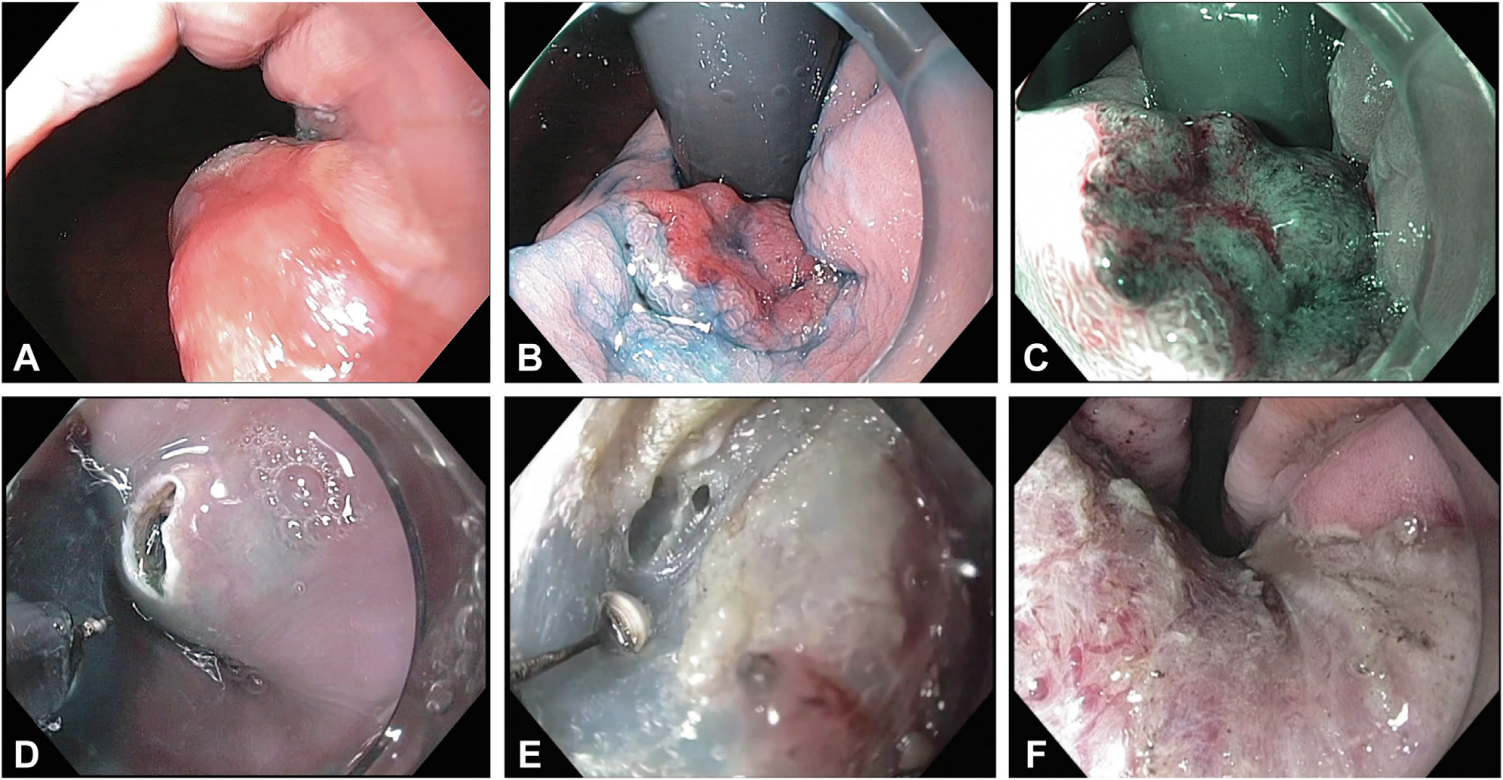
Wide-field endoscopic submucosal dissection steps. **A**, In direct view, an elevated superficial lesion is observed at the gastroesophageal junction, but the distal margin cannot be evaluated. **B**, A 20-mm IIa+IIc lesion and the lesion’s distal margin are seen in the retroflexion view using indigo carmine dye spraying. **C**, Nonmagnifying narrow-band imaging revealed a clear demarcation line and irregular vascular and structural patterns. **D**, The esophageal mucosal incision is made 5 mm from the most proximal marking dot. **E**, Submucosal dissection is performed using the IT-Knife2 in the retroflexion view. **F**, Hemicircumferential mucosal defect after endoscopic submucosal dissection.

A 58-year-old male patient with a body mass index of 36 and a history of chronic GERD underwent an EGD. White-light high-definition examination revealed a C0M3 BE and a 20-mm, IIa+IIc lesion located in the right quadrant of the GEJ at 40 cm from the incisors (Fig. 1A and B). In the direct view, the evaluation of the tumor was technically demanding, and the distal margin could not be evaluated. In the retroflexion view, the lesion’s distal margin was clearly seen in contact with the proximal end of the gastric folds; indigo carmine chromoendoscopy revealed no ulcer, converging folds, or expanded change suggesting a superficial invasion. Nonmagnifying narrow-band imaging revealed a clear demarcation line with irregular vascular and mucosal patterns suggesting a T1a depth of invasion (Fig. 1C). Tumor biopsy revealed an intramucosal adenocarcinoma, and gastric biopsies showed nonatrophic chronic gastritis. The patient was scheduled for a WF-ESD aiming to eradicate the tumor and most of the surrounding non-neoplastic BE.

## Technique

**Figure 2:**
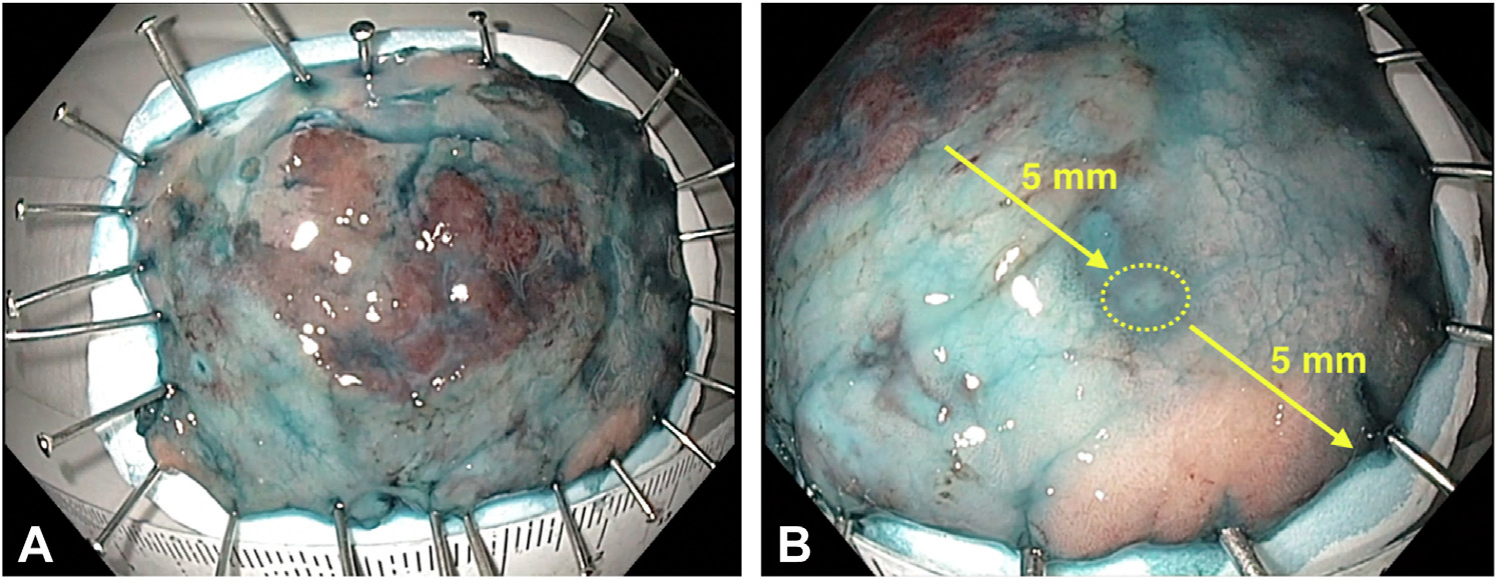
Evaluation of the resected specimen. **A**, The tumor and the specimen were 20 mm and 48 mm, respectively. **B**, The distance between the tumor edge and the whole radial specimen’s margin was at least 10 mm.

**Figure 3:**
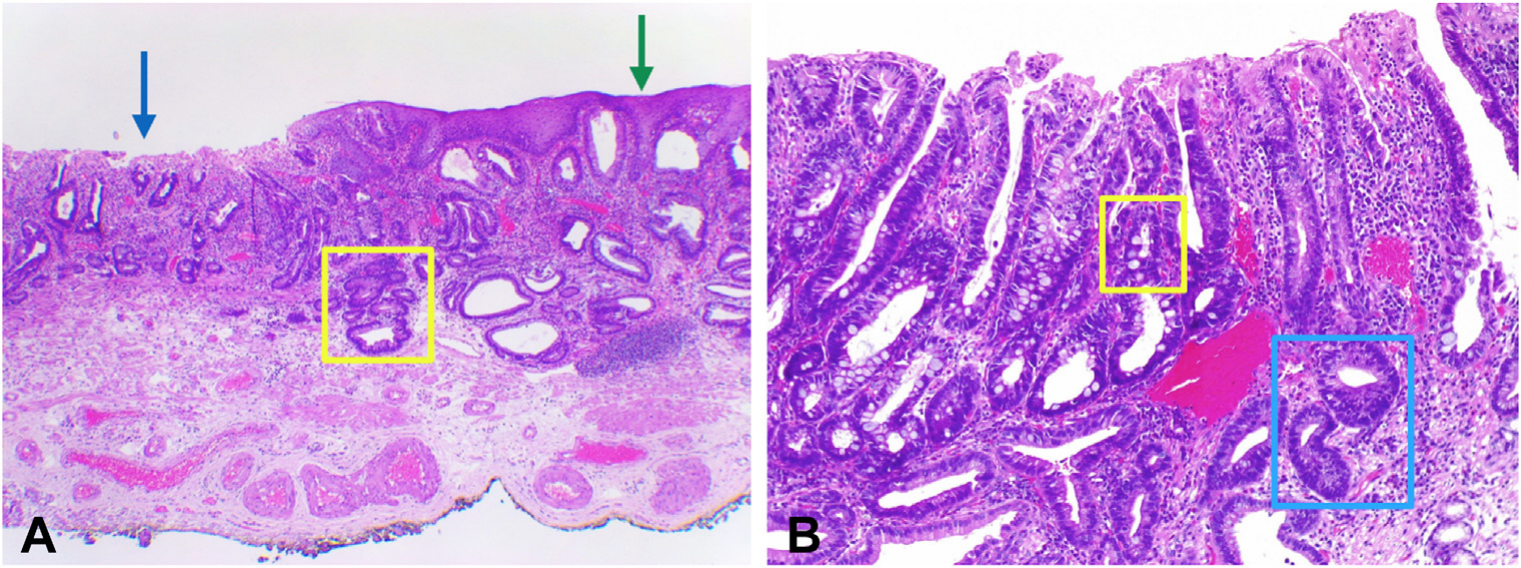
Histopathological analysis. **A**, Panoramic hematoxylin and eosin staining revealed an intramucosal adenocarcinoma (*blue arrow*) without lymphovascular invasion. Normal esophageal squamous epithelium (*green arrow*) and low and high grade dysplastic glands in contact with the muscular mucosae (*yellow square*). **B**, High-power field. Goblet cells are seen confirming the presence of intestinal metaplasia (*yellow square*). Hyperchromatism, pseudostratification, nuclear atypia, and no lymphovascular invasion are observed (*blue square*).

The procedure was performed with the patient under intravenous sedation using an H-180J gastroscope and an EXERA II video processor (Olympus, Tokyo, Japan), an ERBE VIO 300D electrosurgical generator (ERBE Elektromedizin, Tubingen, Germany), a distal transparent cap (Olympus, Tokyo, Japan) and carbon dioxide insufflation. Esophageal and gastric marking dots were placed in direct and retroflex views, respectively, at least 5 mm from the lesion’s margin using a conventional needle knife. A mixture of normal saline, indigo carmine dye, and epinephrine was used as the submucosal (SM) lifting solution. In direct view, a mucosal incision was made over the squamocolumnar esophageal epithelium at approximately 5 mm from the most proximal marking dot (Fig. 1D). Then, the entire procedure continued in the retroflex view and initiated with the circumferential incision using an IT-Knife2 (KD-611L, Olympus, Tokyo, Japan), maintaining a distance of at least 5 mm from all marking dots. The submucosal layer was dissected using endocut mode, effect 2, from the stomach to the esophagus and from the left and right lesion’s sides toward the center of the lesion until en bloc resection was achieved (Fig. 1E and F). By this approach, the counter traction force of the gravity lifted the lesion allowing a clear visual of the dissection plane. Small vessels were selectively coagulated by mainly using the short blades located at the back of the IT-Knife2. The bleeding source from small vessels was identified by pouring a few drops of water over the pool of blood using the distal tip nozzle for lens cleaning and then was controlled using forced coagulation mode, effect 2. Although mild SM fibrosis was seen during the last part of the dissection, an en bloc hemicircumferential resection was achieved without adverse events in 42 minutes. The size of the resected specimen was 48 mm, and the distance between the tumor edge and the whole radial specimen’s margin was at least 10 mm (Fig. 2A and B). The WF-ESD steps accompanied by technical approaches and the endoscopic view are summarized in Table 1. The postoperative course was uneventful and the patient was discharged the same day with oral esomeprazole. Histopathology revealed a free lateral margin well-differentiated intramucosal adenocarcinoma without lymphovascular invasion and associated intestinal metaplasia (IM) (Fig. 3A and B). Follow-up endoscopy showed a clean ESD scar, no stenosis, and 5-mm tongues of BE located at the left quadrant. Complete remission of IM was achieved after a single radiofrequency ablation (RFA) session using the HALO^90^ RFA device.^6^

**Table 1:** Wide-field endoscopic submucosal dissection steps and technical approaches

Steps	Technical approach	Endoscopic view
Marking	∼5 mm from the tumor margin (needle knife)	Esophagus: direct view Stomach: retroflex view
Submucosal injection	Mixed solution of normal saline, epinephrine, indigo carmine dye, and hyaluronic acid (injection needle)	Esophagus: direct view Stomach: retroflex view
Esophageal mucosal pre-cut	Shallow incision after enough submucosal lifting (needle knife)	Direct view
Circumferential incision	∼5 mm from marking dots; include surrounding Barrett's esophagus and tongues (IT-Knife2)	Retroflexion view
Submucosal dissection	Fine lateral movements left to right and right to left from the stomach to the esophagus (IT-Knife2)	Retroflexion view
Hemostasia	Precise location of a bleeding site by pouring water throughout the dial tip lens cleaning nozzle. (IT-Knife2)	Retroflexion view

## Discussion

In contrast to the poor visualization, stability, and maneuverability inherent to ESD conducted using the conventional direct view approach, the retroflexion view approach allowed the resection of a wide margin of gastric mucosa, facilitating an en bloc R0 resection in a relatively short time. This is noteworthy because the lesion was located at the right quadrant of the GEJ, and the patient was maintained in the left lateral position during the entire procedure. As a result, the counter-traction force of gravity pulled up the lesion allowing a better vision and minimizing the risk of perforation without the use of external traction methods. As reported by the authors here^7^ and other experts,^8^^,^^9^ when performing marking and mucosal cutting during some ESD procedures, a conventional needle knife is often the preferred tool over other dedicated ESD accessories. Advantages of using a needle knife include reusability, blade length adjustment, and low cost. Reports have addressed using the same ESD knife for SM dissection and minor bleeding control.^10^^,^^11^ In particular, the short blades of the IT-knife2 provide the ability to coagulate small blood vessels, control mild bleeding, and facilitate a faster dissection.^9^ This targeted approach to vessels eliminates the need for hemostatic forceps in all instances, reducing the operator's effort and the duration of the procedure. Although WF-ESD is a challenging procedure requiring training and expertise, it is a promising alternative to reduce the high rate of positive lateral margins when performing ESD for BA of the GEJ. Contrary to circumferential ESD for Barrett's neoplasia^12^ and ESD cases encompassing ≥75% of the esophageal circumference in which oral or local steroids are indicated to prevent stricture,^5^^,^^13^ prophylaxis using steroids was not indicated in the present hemicircumferential ESD case. To reduce the likelihood of recurrent dysplasia and metachronous cancer, current BE management strategies recommend endoscopic resection of visible neoplasia followed by RFA sessions until complete eradication of intestinal metaplasia is achieved.^14^ Further studies are warranted to demonstrate whether extensive removal of metaplastic tissue by using WF-ESD might increase the likelihood of achieving complete eradication of intestinal metaplasia with fewer RFA sessions.

## Disclosure

All authors disclosed no financial relationships. This work was supported in part by a grant in aid for the Comprehensive Strategy to Control Cancer in the Americas from the Emura Foundation for the Promotion of Cancer Research, ID No. 0223-17.

## Supplementary data

Video 1 Wide-field ESD for Barrett's adenocarcinoma.
